# Assessment of kinship detection using RNA-seq data

**DOI:** 10.1093/nar/gkz776

**Published:** 2019-09-10

**Authors:** Natalia Blay, Eduard Casas, Iván Galván-Femenía, Jan Graffelman, Rafael de Cid, Tanya Vavouri

**Affiliations:** 1 Program for Predictive and Personalized Medicine of Cancer, Germans Trias i Pujol Research Institute (PMPPC-IGTP), Badalona 08916, Spain; 2 Josep Carreras Leukaemia Research Institute (IJC), Campus ICO-Germans Trias i Pujol, Universitat Autònoma de Barcelona, Badalona 08916, Spain; 3 Masters Programme in Bioinformatics and Biostatistics, Universitat Oberta de Catalunya (UOC), Barcelona 08035, Spain; 4 Doctoral Programme in Biomedicine, Universitat de Barcelona, Barcelona 08007, Spain; 5 Genomes for Life - GCAT lab Group - Germans Trias i Pujol Research Institute, Can Ruti Campus, Ctra de Can Ruti, Camí de les Escoles s/n, Badalona, Barcelona 08916, Spain; 6 Department of Statistics and Operations Research Universitat Politècnica de Catalunya, Barcelona 08028, Spain; 7 Department of Biostatistics, University of Washington, Seattle, WA 98105-946, USA

## Abstract

Analysis of RNA sequencing (RNA-seq) data from related individuals is widely used in clinical and molecular genetics studies. Prediction of kinship from RNA-seq data would be useful for confirming the expected relationships in family based studies and for highlighting samples from related individuals in case-control or population based studies. Currently, reconstruction of pedigrees is largely based on SNPs or microsatellites, obtained from genotyping arrays, whole genome sequencing and whole exome sequencing. Potential problems with using RNA-seq data for kinship detection are the low proportion of the genome that it covers, the highly skewed coverage of exons of different genes depending on expression level and allele-specific expression. In this study we assess the use of RNA-seq data to detect kinship between individuals, through pairwise identity by descent (IBD) estimates. First, we obtained high quality SNPs after successive filters to minimize the effects due to allelic imbalance as well as errors in sequencing, mapping and genotyping. Then, we used these SNPs to calculate pairwise IBD estimates. By analysing both real and simulated RNA-seq data we show that it is possible to identify up to second degree relationships using RNA-seq data of even low to moderate sequencing depth.

## INTRODUCTION

RNA sequencing is used in genetics for a variety of purposes such as to test the heritability of gene expression traits (1), to search for mutations causing mendelian disorders (2), to understand the effects of disease-associated mutations (e.g. (3)), to study the mechanisms of epigenetic inheritance of phenotypic traits (4), among others. In these studies, multiple RNA samples are extracted from different related or unrelated individuals and they are processed in parallel. Knowing whether samples are from related individuals and their exact relationship is important. For example, in family studies, matching samples to individuals in a pedigree allows the correct association of phenotypic traits to expression patterns. In case-control gene expression studies, the inclusion of samples from related individuals leads to a mis-specified covariance structure and therefore an inflated type-1 error rate when testing for association. Knowing the true relatedness of the samples allows researchers to remove unwanted related samples or use a method that models expression taking into account relatedness. In population studies, biased recruitment schemes can enrich datasets with cryptic relationships (5). This is a common scenario in association analyses and it could also happen for RNA-seq collections from unrelated individuals. Currently, researchers check the correctness of RNA-seq sample relatedness indirectly because it is so far unclear whether kinship can be detected directly from RNA-seq data.

During sample and data processing, sample mislabeling can occur due to human error. This is detrimental for downstream analysis, especially for family studies, and has been estimated to affect at least 4% of published samples (6,7). Given the importance and pervasiveness of this problem, there are numerous programs and methodologies that deal with sample mislabeling in sequencing data by comparing paired samples from the same individual (8–11), comparing the annotated sex with expression of sex-specific genes (12) or using a heuristic data perturbation strategy (7). From genotyping, whole genome sequencing (WGS) and whole exome sequencing (WES) datasets, there are numerous methods that can predict kinship from single nucleotide polymorphisms (SNPs) and confirm that samples correspond to the labelled individuals in a pedigree or that they are genetically unrelated. However, none of these methods sufficiently address labeling mix-ups or unreported relatedness of samples when only RNA-seq data is available, for each individual. Furthermore, for researchers interested in analyzing only expression data, current methods require them to retrieve and process in parallel at least one additional data file (WGS, WES or genotyping array data) per individual, so as to confirm that RNA-seq and DNA sequencing data contain the same nucleotide variants and then use the DNA sequencing data to extract genotypes from which to confirm kinship. Even when available, the retrieval and analysis of WGS or WES just to check for errors in RNA-seq sample relatedness is a cumbersome and often very time consuming task.

There are several methods to predict or confirm familial relationships and build heritability estimates from genetic data. Although these methods were initially based on microsatellites (13) and amplified fragment length polymorphism (AFLPs) (14), nowadays they usually rely on SNPs. Among the different methodologies for kinship detection, we can find methods that exclude impossible parent-offspring relationships (15–17), methods that calculate kinship coefficients and/or identical-by-descent (IBD) probabilities (18,19) and likelihood methods (20,21). RNA-seq data can be used to identify SNPs (22). Yet, the use of RNA-seq data for kinship detection and pedigree reconstruction has not been properly assessed so far. Some of the concerns with using this type of data for kinship detection are the limited number of SNPs adequately covered by reads and allelic imbalance (23,24) that may hinder genotype calling by masking one of the two alleles at many variant positions along the genome.

Here, we report the results of our assessment of the utility of human RNA-seq data for kinship detection and pedigree reconstruction. Our input data is a set of raw RNA-seq data files and a set of known common variant positions. First, we filter both the RNA-seq data and the known variants to obtain reliable predicted genotypes at common variant positions for each RNA sample. We then use the predicted genotypes to detect and represent familial relationships by estimating pairwise probabilities of identity-by-descent (IBD) (18,25). By analysing human empirical as well as simulated RNA-seq data we show that the estimated IBD probabilities allow kinship detection and pedigree reconstruction, detecting up to second degree relationships even with low sequencing depth. We propose kinship detection directly from RNA-seq data as a simple and direct method to detect sample relatedness.

## MATERIALS AND METHODS

### Retrieval of empirical data

**Figure 1.**
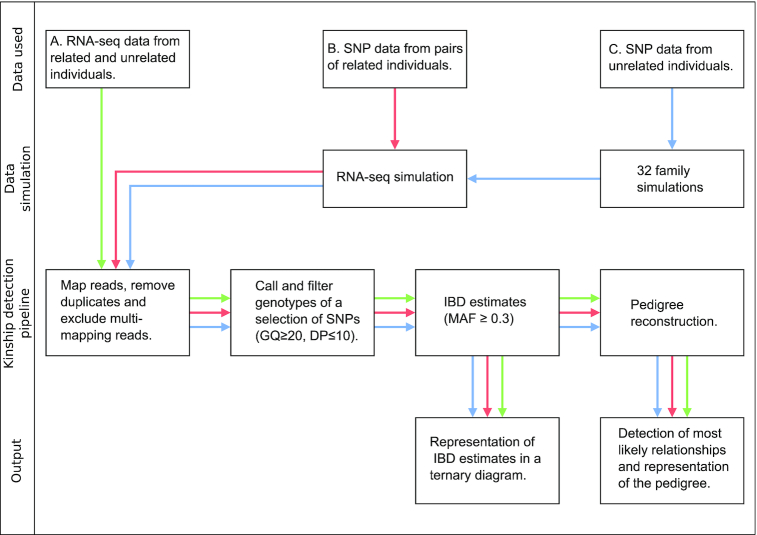
Overview of data workflow for kinship detection and pedigree reconstruction using RNA-seq data. GQ: genotype quality, DP:depth, IBD: identity by descent, MAF: minor allele frequency.

We used three types of empirical data (Figure 1, Supplementary Table S1). First, we used previously published RNA-seq data from four different studies that include transcriptome sequencing from a 17-member, three-generation family (26), transcriptome sequencing from a trio (3), transcriptome sequencing from a pair of first degree relatives and two unrelated individuals (27) as well as targeted transcript sequencing (Ion AmpliSeq, Life Technologies) from 7 unrelated individuals and a pair of siblings (28). Second, we retrieved genetic variation data from six pairs of first and second degree relatives from the CDX population of the 1000 Genomes Project (29). From this data we then simulated RNA-seq reads (the method of simulation of RNA-seq reads is described below). Third, we retrieved genetic variation data from unrelated individuals from the 1000 Genomes Project (29) from which we simulated families (the method of simulation of genotypes of family members is described below) and RNA-seq reads.

### Simulation of genotypes of family members

To simulate the genotypes of family members we used SNP data from the 1000 Genomes Project (29). We selected unrelated individuals as founders and used their haplotype data to simulate the rest of the family. We simulated data according to different pedigrees in order to assess the effect of pedigree complexity. Family simulations were carried out following Mendelian laws and taking into account linkage disequilibrium (assuming 1 cM per Mb) with a custom R script.

### Simulation of RNA-seq reads

To simulate gene expression data from real or simulated individuals, we generated paired-end RNA-seq reads with flux-simulator v1.2.1 (30). We used two genome fasta files per individual (one per haplotype, including the SNPs to the reference genome with GATK v3.8 FastaAlternateReferenceMaker (31)) and the expression profile of B lymphocytes (custom .pro file with RPKMx150 obtained from the founders of CEPH/UTAH family 1463 (26) to get a sequencing depth of 40M reads per individual). We ran the simulation with library preparation and sequencing steps (-ls options) for the first haplotype. For the second haplotype we used the library (.lib file) of the first haplotype and ran only the sequencing step (-s option) to make sure the same genes are expressed in both haplotypes.

### Data filtering, read mapping and kinship detection

**Figure 2.**
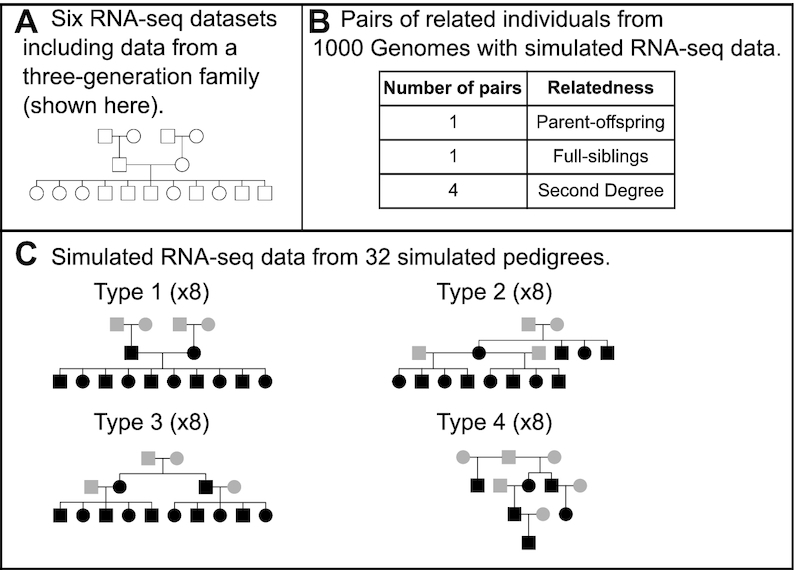
Structure of datasets used for the assessment of kinship detection using RNA-seq data. (**A**) Structure of the extended CEPH/UTAH family 1463 with empirical RNA-seq data (26). Additional empirical RNA-seq datasets are included in Supplementary Figure S2. (**B**) List of real related pairs of individuals with simulated RNA-seq data. (**C**) Simulated families with simulated RNA-seq data (pedigree types 1–4). Real individuals with simulated RNA-seq data are highlighted in grey. Simulated individuals with simulated RNA-seq data are highlighted in black.

We aligned empirical and simulated RNA-seq reads to the reference genome hg19 using HISAT2 v2.1.0 (32). For mapping, we used the HISAT2 index for the human reference genome plus transcripts retrieved from ftp://ftp.ccb.jhu.edu/pub/infphilo/hisat2/data/grch37_tran.tar.gz (accessed: Nov 8, 2018). We then used SAMtools to remove duplicates (command markdup). We retrieved 14.8 million (M) common SNPs from the UCSC Genome Browser (dbSNP Build ID 150) (33). We used BEDtools intersect v2.26.0 (34) to identify and remove 38,572 SNPs in 83 imprinted genes (http://www.geneimprint.org/ accessed: 8 March 2018) and 8.2 M SNPs in repeats (RepeatMasker annotation downloaded from the UCSC Genome Browser), ending up with 6.2 M SNPs. Genotypes were obtained with SAMtools mpileup v1.7 (options -A -q 4 -t AD,DP) (35) and BCFtools call v1.4 (options -m - -O b -f GQ) (36), using uniquely mapping reads only. We used VCFtools v0.1.14 (36) to select only those SNPs with a depth ≥ 10 (option –minDP 10) and a genotype quality ≥ 20 (option –minGQ 20). To assess the accuracy of genotype prediction from RNA-seq data, we retrieved high confidence variant calls for two individuals of CEPH/UTAH family 1463 (the two offspring of the four founders in the pedigree shown in Figure 2A) (37). The effect of the different SNP filtering steps on the number of variants considered for IBD estimation, the accuracy of genotype prediction (for individuals NA12877 and NA12878 of CEPH/UTAH family 1463), kinship detection and pedigree reconstruction is shown in Supplementary Table S2.

To determine pairwise IBD estimates we used PLINK v1.9 (38), considering only autosomal SNPs with a minor allele frequency ≥0.3 (–maf 0.3 option) to obtain an optimal separation between groups. Use of a higher minor allele frequency threshold led to high deviation of Z0, Z1 and Z2 from the theoretical values (Supplementary Figure S1). Representation of pairwise IBD estimates was done in R v3.5.0 (39) with the method described by Galván-Femenía *et al.* (40). Using the IBD estimates obtained in the previous step, we used PRIMUS v1.9.0 (41) to predict pairwise relationships and to reconstruct the whole pedigree, considering as unrelated those pairs of individuals with a coefficient of relatedness lower than 0.2 (option -t 0.2). Sex data was also provided. The sex of the individuals from the real data was inferred from counting reads mapping on the Y chromosome and compared against the reported sex.

## RESULTS

### Analysis workflow for kinship detection using RNA-seq from related individuals

To assess how feasible it is to detect kinship using RNA-seq data we used three types of data (Figures 1 and 2). First, we used six previously published sets of RNA-seq data from related and unrelated individuals from four different studies that include samples from an extended three-generation human family (26) (Figure 2A). Second, we used available SNP data from six pairs of first and second degree relatives from the CDX population of the 1000 Genomes Project (29) from which we simulated RNA-seq data (Figure 2B). Third, we used available SNP data from unrelated individuals from the 1000 Genomes Project (29) from which we simulated the genotypes of family members and the corresponding RNA-seq samples (Figure 2C). We simulated four types of pedigrees to assess the effect of pedigree complexity. After data retrieval or simulation, we used the same analysis workflow which in brief consisted of mapping and filtering RNA-seq reads, calling and filtering genotypes of a set of filtered SNPs, calculating IBD estimates and finally visualizing the IBD estimates in ternary diagrams or providing the IBD estimates to the software PRIMUS for prediction of pairwise relationships and pedigree reconstruction.

### Kinship detection using empirical human RNA-seq data

We mapped and filtered the raw RNA-seq data from B-lymphocytes from members of the CEPH/UTAH family 1463 (26) and in parallel filtered the initial set of known common human variants as described in the methods. Reads were mapped to the reference human genome. This step excluded on average 12% of the initial reads (range 6–24%, depending on the individual) that could not be mapped. We then removed PCR duplicates as they can potentially amplify sequencing errors. This step led to the removal of an average of 21% of the reads (range 17–30%). We also removed multi-mapping reads to avoid mapping errors, excluding on average 4.6% of the reads (range 4.1–5.3%). We filtered the known common human variants; we removed SNPs in repeats and in imprinted genes (55% and 0.26% of SNPs excluded, respectively) that could bias genotyping. Having obtained a filtered set of RNA-seq reads (a mean of 34M reads per individual) and SNPs (6.2 M SNPs), we proceeded to call and filter genotypes, obtaining 633,636 SNPs that were covered by reads in at least one individual. To exclude those SNPs with low coverage we selected a minimum depth of 10 reads per SNP, and to avoid genotyping errors we filtered out those SNPs with a genotype quality lower than 20, which means an error rate of 0.01 (in this step we excluded 76% of the SNPs). Finally, we considered those SNPs with a minor allele frequency of at least 0.3 (excluding 89.5% of the SNPs). We ended the filtering process with 16,004 SNPs present in at least one individual. The number of SNPs shared between pairs of individuals that were used for downstream analyses was between 5,657 and 8,600, depending on the number of missing genotypes observed for each pair of individuals. High confidence variant calls are available for two individuals (individuals with identifiers NA12877 and NA12878) of the CEPH/UTAH family 1463 that we have analysed. These high confidence variant calls agreed with those predicted from RNA-seq data using our pipeline at 99.3% and 98.8% of the common variant positions that passed all filters.

**Figure 3.**
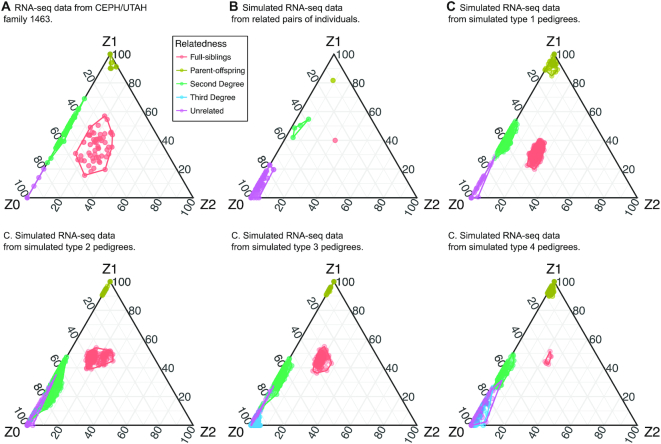
Ternary diagrams of IBD estimates for (**A**) CEPH/UTAH family 1463, (**B**) simulated RNA-seq data from real related pairs of individuals and (**C**) simulated RNA-seq data from simulated related individuals from different pedigree types (type 1–type 4). Note that in ternary diagrams data points that have very similar Z0, Z1 and Z2 values overlap and may not be visible. Ternary diagrams for five additional empirical RNA-seq datasets are shown in Supplementary Figure S2.

We used ternary diagrams to visualize the estimated probabilities of sharing 0, 1 and 2 IBD alleles (referred here as Z0, Z1 and Z2 respectively) meaning that none, only one or both alleles of the pair are inherited from the same recent common ancestor. In ternary diagrams, the coordinates of each data point are the three IBD estimates for each pair of individuals in the dataset. We observed that the different relationships were grouped around their theoretical values with no overlap between them. As expected, parent-offspring pairs are clustered at the Z1 corner (meaning that they share 1 IBD allele at all SNPs), unrelated individuals at the Z0 corner (they do not share any IBD alleles), second degree relationships on the Z0-Z1 axis (they share 1 IBD allele at half of their SNPs) and full-siblings in the middle of the graph (they can share from 0 to 2 IBD alleles per SNP) (Figure 3A, Supplementary Table S3).

The actual pedigree was also correctly reconstructed with PRIMUS (41) from the estimated IBD values. In addition to the top scoring pedigree, which in this case corresponds to the correct one, PRIMUS also reports the predicted pairwise relationships. In this case it did not correctly identify some of the pairwise relationships; the mis-identified pairs were 2% of full sibling pairs (one pair was classified as second degree relatives), 32% of second degree pairs and 9% of unrelated pairs of individuals.

In addition to the data from the 17-member family, we assessed kinship detection for a family trio (mother, father, daughter) using transcriptome sequencing data from cardiomyocytes differentiated from induced pluripotent stem cells (3), for a parent-offspring pair and unrelated individuals using transcriptome sequencing data from whole blood and peripheral blood mononuclear cells (27) and for a pair of siblings and seven unrelated individuals using targeted RNA sequencing from naive CD4+ T-cells and LPS-stimulated monocytes (28) (Supplementary Figure S2A–E). For whole transcriptome sequencing, the number of SNPs used for pairwise comparisons and IBD estimation ranged from 1,311 to 5,876 SNPs, depending on the pair of individuals analysed. For targeted RNA-seq samples, generated using the Ion AmpliSeq Transcriptome Human Gene Expression Kit (Life Technologies) that targets on average 150 bp per human gene, the number of SNPs covered and passing all filters were significantly fewer, as expected. Specifically, from Ion AmpliSeq data, the number of SNPs used for pairwise comparisons and IBD estimation ranged between 95 and 193. Samples from related individuals (parent-offspring pairs and siblings) are clearly separated from replicates (tightly clustering at the Z2 corner) and from unrelated individuals in all datasets, including in the targeted RNA-seq datasets (Supplementary Figure S2). In these datasets, although samples from unrelated individuals are clearly separated from samples from first-degree relatives, their IBD estimates are further from their theoretical values, something that was improved by removing replicates (data not shown). Relatedness was correctly inferred by PRIMUS, however for the transcriptome sequencing data from whole blood and the targeted RNA-seq data from peripheral blood mononuclear cells other high scoring pedigrees were also suggested.

According to these results, we can conclude that kinship detection is possible with RNA-seq data, allowing us to detect and represent first and second degree relationships. Although the sensitivity decreases as the degree of relatedness increases, pedigree reconstruction is robust. As an indication of the time required to perform kinship detection, the full RNA-seq analysis of nine samples from the trio (father, mother and offspring with three replicates per individual from (3)) took 110 min using an Intel(R) Xeon(R) W-2145 CPU 3.70GHz workstation with eight cores and 64GB of memory (parallel processing was used only for mapping reads). This includes the time needed to map the raw reads (∼45 min).

### Impact of sequencing depth on kinship detection

The sequencing depth of the real RNA-seq data from the extended human family ranged from 37M to 67M reads, with a mean depth of 52M. To understand the effect of sequencing depth on kinship detection, we randomly subsampled different numbers of reads from the FASTQ input files. We subsampled 37M reads (the number of reads for the individual with the lowest coverage) to homogenize the sequencing depth for all individuals and then also 30M, 20M, 10M, 8M, 6M, 4M and 2M reads from each sample to determine the minimum sequencing depth for kinship detection and pedigree reconstruction.

**Figure 4.**
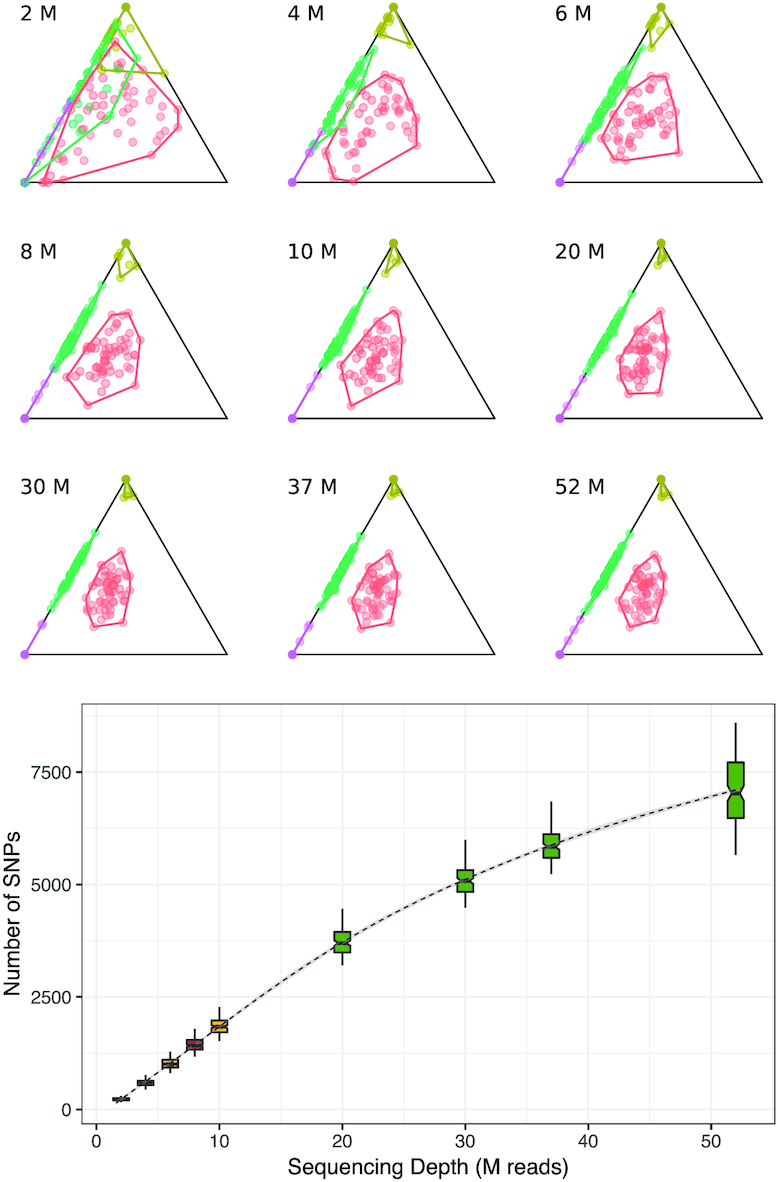
Ternary diagrams of IBD estimates for each sequencing depth (top) and number of SNPs used for pairwise comparison per sequencing depth (bottom). Green color indicates that the pedigree was correctly reconstructed, yellow colour indicates that there was no overlapping between groups but the pedigree reconstruction was not possible, and red color indicates that there was an overlapping between groups. The box with a sequencing depth of 52 is the one for the original data, being the mean for the different sequencing depths (from 37M to 67M reads).

Visual inspection of estimated probabilities using ternary diagrams (Figure 4) revealed that there was no overlap between second degree relatives and unrelated individuals when using RNA-seq data with 52M, 37M, 30M, 20M, 10M and 6M reads. In the simulation of 8M reads we observed a slight overlap between second degree relatives and unrelated individuals. In the simulation of 4M reads, there was overlap between second degree and unrelated pairs of individuals and also between second degree and full siblings. With 2M reads all groups overlapped except parent-offspring and unrelated pairs of individuals.

Pedigree reconstruction was only possible for RNA-seq data with 52M, 37M, 30M and 20M reads because, when decreasing the sequencing depth, the probability to share one IBD allele (Z1) of some full siblings decreased way below the expected values (e.g. Z0 = 0.6741, Z1 = 0.0712, Z2 = 0.2547).

Taken together, these results demonstrate that sequencing depth affects the number of SNPs used for pairwise comparison, but overall its effect on kinship detection is small, providing acceptable IBD estimates (no overlapping between relationships) even with 6M reads, where the number of SNPs is as low as 811–1,282 (Figure 4). As IBD estimates with 8M reads were not as good, we conclude that the minimum sequencing depth to be able to detect kinship in RNA-seq data through IBD probabilities is 10M reads. In contrast, the minimum sequencing depth to be able to reconstruct the correct pedigree is 20M reads.

### Kinship detection using empirical genotypes of related individuals with simulated RNA-seq data

To further assess the feasibility of estimating relationships from RNA-seq samples from related individuals, we simulated RNA-seq data from real related individuals with known genotypes. We used variation data from six pairs of first and second degree relatives from the CDX population of the 1000 Genomes Project (Figure 2B). We simulated paired-end RNA-seq reads with the expression profile of B-lymphocytes. We obtained this gene expression profile from the mean of the number of reads mapped to a gene per gene length in kilobases per million mapped reads (RPKM) of the founders of the CEPH/UTAH family 1463. Then, we multiplied those values by a factor of 150 to simulate the desired expression profile with a sequencing depth of 40M reads. As expected, the simulated RNA-seq data is highly correlated with the real data (Supplementary Figure S3)—Pearson's correlation estimates are on average 0.918 (range 0.913–0.925). We used the same workflow described for the empirical RNA-seq data. The resulting number of SNPs used for the pairwise comparisons was between 4,501 and 4,632.

In agreement with the results obtained from the real RNA-seq data, visual inspection of estimated probabilities in ternary diagrams revealed that all relationships are clearly separated (Figure 3B). Similarly, the pedigree reconstruction program was able to correctly classify and represent all relationships in a pedigree. We conclude that kinship detection using RNA-seq data is possible using different datasets; for entire families as well as for pairs of related individuals.

### Kinship detection using simulated genotypes of family members with simulated RNA-seq data

We then asked whether the complexity of the pedigree could affect kinship detection and pedigree reconstruction. To address this, we generated additional RNA-seq data from different types of pedigrees, this time simulating both gene expression and also the genotypes of individuals in the pedigrees. We used genotypes of real unrelated individuals from the 1000 Genomes Project as founders (29) (Figure 2C, genotypes of real individuals shown in gray, simulated genotypes of offspring shown in black). We simulated families using data from eight different populations from the 1000 Genomes Project: GBR, KHV, IBS, LWK, CLM, CDX, PEL and ACB; and four pedigree structures including different degrees of relationship (Figure 2C):

Type 1: A 16-member pedigree with the same relationships as CEPH/UTAH family 1463.
Type 2: A 16-member pedigree with all first and second degree relationships from Supplementary Table S3.
Type 3: A 16-member pedigree with first, second and third degree relationships.
Type 4: A 12-member pedigree with all first, second and third degree relationships from Supplementary Table S3.

**Figure 5.**
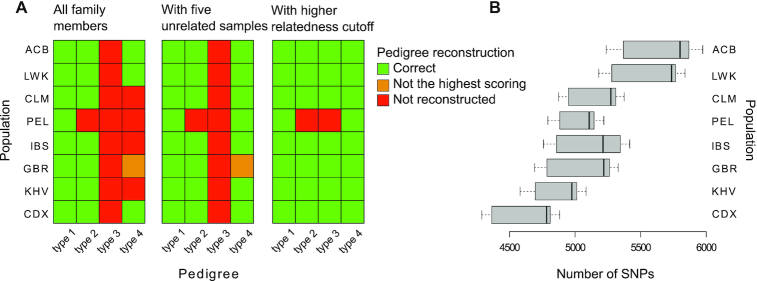
Pedigree reconstruction using RNA-seq data. (**A**) The matrices show the correctly reconstructed simulated pedigrees with our default workflow (left panel), with five additional unrelated individuals (middle panel) and with a higher relatedness cutoff (0.375 instead of 0.2) (right panel). (**B**) The boxplots summarize the distributions of the number of SNPs used for pairwise comparisons and IBD estimation for each pair of individuals in each simulated pedigree.

We made 32 simulations, one for each pedigree type and population. Sex was not simulated as sex chromosomes are not used for IBD estimation. After data filtering, we observed that the number of SNPs used for pairwise comparison was higher in African populations (ACB and LWK) ranging from 5,180 to 5,974 and lower in Asian populations (KHV and CDX) ranging from 4,289 to 5,084 (Figure 5B). To further assess the accuracy of our genotype prediction pipeline, we compared the genotypes of individuals in a simulated pedigree (type 4 pedigree with founders from IBS population) to the predicted genotypes and found them to agree at 98.3–99.2% of the common variant sites that passed all filters.

Visual inspection of IBD probabilities (Figure 3C) revealed that first degree relationships were clearly separated from other relationships, second degree relationships were separated from unrelated individuals in all simple pedigrees (type 1 simulated pedigrees) and some type 2, 3 and 4 simulated pedigrees. In contrast, third degree relationships overlapped unrelated pairs of individuals in all pedigrees where they were present (Figure 3C, pedigree type 3–type 4).

Reconstruction of the actual pedigree was possible for all simulated type 1 pedigrees, seven type 2 pedigrees and four type 4 pedigrees (one of them was not the top scoring one, but the fourth in the PRIMUS output), but for none of the type 3 pedigrees (Figure 5A, left panel). Although PRIMUS was not able to reconstruct all the simulated pedigrees, it was able to identify all first degree relatives, 35% of second degree relatives (in type 1 pedigrees this percentage was 68%), 12% of third degree relatives, and 93% of unrelated individuals (Supplementary Figure S4).

We conclude that family structure affects kinship detection, providing good IBD estimates for simple families with up to second degree relatives, but in pedigrees with third degree relatives there is overlap between different relationships. Accordingly, entire pedigree reconstruction works better for simple families, getting worse when the number of third degree related pairs increases (type 3 pedigree has 25 pairs of third degree related individuals while type 4 pedigree has six). Population specific differences in genetic variability lead to different number of SNPs that pass all filters, however these differences do not affect the results.

We wondered whether the addition of unrelated individuals would improve kinship detection. Presumably, they include more variability and make related individuals look more similar to each other. To test this, we added five unrelated individuals from the same population to each simulated family. The result was just as good for all families that were previously correctly reconstructed but it improved for four families, reconstructing correctly all type 1, seven type 2, all type 4 pedigrees but none of type 3 (Figure 5A, Middle Panel). We conclude that adding data from unrelated individuals would be useful in those cases where the reconstruction is not possible.

We asked whether it was possible to correctly predict the pedigree structure using only the most related individuals in complex pedigrees by using a stricter relatedness cutoff. We reran PRIMUS with a higher relatedness cutoff (option -t 0.375, instead of the previously used -t 0.2) and found that type 1 and type 2 pedigrees were reconstructed equally well but also that seven out of eight type 3 pedigrees and all type 4 pedigrees could also be correctly reconstructed (Figure 5A, right panel). We conclude that a more restrictive relatedness cutoff works better than adding unrelated individuals in those cases where most or all of the individuals of the pedigree are connected through a first degree relative.

Last, we tested whether IBD estimation and kinship detection could be used to identify RNA-seq samples from related individuals in a dataset consisting mostly of samples from unrelated individuals. To test this we retrieved genotypes for twenty unrelated individuals of the IBS population from the 1000 Genomes Project (29) (Supplementary Table S1) and we used them to simulate RNA-seq data as done previously. We then generated five different RNA-seq datasets, each consisting of the twenty simulated RNA-seq samples from unrelated individuals from the 1000 Genomes Project (29) and a pair of the simulated RNA-seq samples from the previously generated type 4 pedigree (with IBS genotypes as founders) (Supplementary Figure S5). The estimated IBD probabilities clearly separated a pair of RNA-seq samples from second degree relatives (grandmother-grandson) from samples from twenty unrelated individuals from the same population. Samples from third degree relatives (great-grandmother and great-grandson and first cousins) had estimated IBD probabilities that were marginally separated from those of unrelated individuals. Samples from fourth degree relatives were indistinguishable from those from unrelated individuals. We tested whether more stringent filtering of variants by removing variants falling in segmental duplications would improve the separation of third and fourth degree relatives from unrelated individuals but this was not the case (Supplementary Figure S6). We conclude that IBD probabilities estimated from RNA-seq data can be used to confidently identify samples from up to second degree relatives and possibly flag samples from third degree relatives within larger RNA-seq datasets from unrelated individuals.

## DISCUSSION

We have shown here that kinship detection based on estimates of identity by descent probabilities using RNA-seq data is possible allowing the detection of up to second degree relationships. In addition, we have shown that the actual pedigrees can be fully reconstructed and, although pedigree reconstruction works better for simple pedigrees, some pedigrees with third degree relatives can also be reconstructed. Furthermore, using simulations, we have shown that the ability to detect kinship between individuals is not limited by RNA-seq sequencing depth since it is still possible at a depth significantly below what is considered acceptable for gene expression analyses (usually 50M reads). Furthermore, we have shown that samples from first degree relatives can be distinguished from replicates and samples from unrelated individuals even with targeted RNA-seq data. Reconstruction of full pedigrees requires higher sequencing depth but is still possible with at least 20M reads.

An issue that remains to be investigated in future studies is whether gene expression data from other species would provide similar results to the ones from humans. Genetic variability among different individuals from a particular species or a particular population could play an important role in kinship detection with higher genetic diversity between individuals leading to better results.

In conclusion, we have shown here that RNA-seq data can be used to identify samples from closely related individuals using estimated identity by descent probabilities calculated from predicted genotypes at common variant positions. It turns out that the effect of calling genotypes correctly at the single nucleotide level due to allele-specific expression is not large enough to impede kinship detection from IBD estimates. We therefore recommend the estimation of IBD probabilities and visualization of the clustering of samples in ternary graphs or the use of pedigree reconstruction programs such as PRIMUS as a quality control step in studies that generate multiple RNA-seq samples from related individuals and population based studies.

## DATA AVAILABILITY

The scripts used for kinship detection and pedigree reconstruction are available through the GitHub repository http://github.com/vavouri-lab/RNA-kinship

## Supplementary Material

gkz776_Supplemental_File
