# Supplementary material for: Assessment of kinship detection using RNA-seq data
Source: Nucleic Acids Res. 2019 Sep 10;47(21):e136. doi: 10.1093/nar/gkz776 (PMC6868348; doi:10.1093/nar/gkz776)
Supplement: gkz776_Supplemental_File [file gkz776_supplemental_file.pdf]

Supplementary Material for

Assessment of kinship detection using RNA-seq data

Authors

Natalia Blay[1,2,3], Eduard Casas[1,2,4], Iván Galván-Femenía[1,5], Jan Graffelman[6,7], Rafael de Cid[1,5], Tanya Vavouri[1,2]

Affiliations

1. Program for Predictive and Personalized Medicine of Cancer, Germans Trias i Pujol Research Institute (PMPPC-IGTP), Badalona, Spain
2. Josep Carreras Leukaemia Research Institute (IJC), Campus ICO-Germans Trias i Pujol, Universitat Autònoma de Barcelona, Badalona, Spain
3. Masters Programme in Bioinformatics and Biostatistics, Universitat Oberta de Catalunya (UOC), Barcelona, Spain
4. Doctoral Programme in Biomedicine, Universitat de Barcelona, Barcelona, Spain
5. Genomes for Life - GCAT lab Group - Germans Trias i Pujol Research Institute, Can Ruti Campus, Ctra de Can Ruti, Camí de les Escoles s/n, Badalona, Barcelona, Spain.
6. Department of Statistics and Operations Research Universitat Politècnica de Catalunya, Barcelona, Spain.
7. Department of Biostatistics, University of Washington, Seattle, USA.

Corresponding author: Tanya Vavouri tvavouri@carrerasresearch.org

This PDF file includes:

Supplementary Table 1

Supplementary Table 2

Supplementary Table 3

Supplementary Figure 1

Supplementary Figure 2

Supplementary Figure 3

Supplementary Figure 4

Supplementary Figure 5

Supplementary Figure 6

Supplementary Table 1. Source and identifiers of publicly available datasets used in the manuscript.

Data type	*Identifier / accession #*	*Reference*	*Usage in the manuscript*
RNA-seq	SRR1258217<br>SRR1258218<br>SRR1258219<br>SRR1258220<br>SRR1258221<br>SRR1258222<br>SRR1258223<br>SRR1258224<br>SRR1258225<br>SRR1258226<br>SRR1258227<br>SRR1258228<br>SRR1258229<br>SRR1258230<br>SRR1258231<br>SRR1258232<br>SRR1258233	Li *et al.*, Am J Hum Genet, 2014.<br><br>Data retrieved from Gene Expression Omnibus record GSE56961.	Kinship detection using RNA-seq data from the 17-member CEPH-UTAH family 1463.
RNA-seq	SRR9070085<br>SRR9070086<br>SRR9070087<br>SRR9070088<br>SRR9070089<br>SRR9070090<br>SRR9070091<br>SRR9070092<br>SRR9070093	Gifford *et al.* Science, 2019.<br><br>Data retrieved from Gene Expression Omnibus record GSE131322.	Kinship detection using RNA-seq data from iPSC-derived cardiomyocytes from a trio. Three replicates per sample.
RNA-seq	SRR7739176<br>SRR7739177<br>SRR7739178<br>SRR7739179<br>SRR7739180<br>SRR7739181<br>SRR7739182<br>SRR7739183<br>SRR7739184<br>SRR7739185<br>SRR7739186	Oda *et al.* Front Immunol 2019.<br><br>Data retrieved from Gene Expression Omnibus record GSE118901	Kinship detection using RNA-seq data from whole blood and peripheral blood mononuclear cells from 1 parent, 1 offspring and 3 unrelated individual with 1-2 replicates per sample.
RNA-seq	SRR6926729<br>SRR6926730<br>SRR6926731<br>SRR6926732<br>SRR6926733<br>SRR6926734<br>SRR6926735<br>SRR6926736<br>SRR6926737<br>SRR6926738<br>SRR6926739	Boutboul *et al* J Clin Invest 2018.<br><br>Data retrieved from Gene Expression Omnibus records GSE112706 and GSE112707	Kinship detection using AmpliSeq (targeted RNA-seq) data from Naive CD4+ cells and LPS-stimulated monocytes from 8 unrelated individuals and two siblings with 2 replicates per sample.

	SRR6926740<br>SRR6926741<br>SRR6926742<br>SRR6926743<br>SRR6926744<br><br>SRR6926281<br>SRR6926282<br>SRR6926283<br>SRR6926284<br>SRR6926285<br>SRR6926286<br>SRR6926287<br>SRR6926288<br>SRR6926289<br>SRR6926290<br>SRR6926291<br>SRR6926292<br>SRR6926293<br>SRR6926294<br>SRR6926295<br>SRR6926296<br>SRR6926297<br>SRR6926298		
Genotypes	NA12877<br>NA12878	Eberle, MA *et al.* Genome Res, 2017	Estimation of genotype prediction accuracy.
Genotypes	HG02363<br>HG02372<br>HG02377<br>HG02381<br>HG02387<br>HG02388<br>HG02250<br>HG02353<br>HG02371<br>HG02373<br>HG02375<br>HG02386	The 1000 Genomes Project Consortium, Nature, 2015.	Kinship detection using simulated RNA-seq data from genotypes from **related** individuals (**CDX** population).
Genotypes	HG00096<br>HG00097<br>HG00099<br>HG00100<br>HG00101<br>HG00102<br>HG00231<br>HG00232<br>HG00233<br>HG00234<br>HG00235	The 1000 Genomes Project Consortium, Nature, 2015.	Kinship detection using simulated RNA-seq data from genotypes from **unrelated** individuals (**GBR** population).
Genotypes	HG02133<br>HG02134<br>HG02136<br>HG02137	The 1000 Genomes Project Consortium, Nature, 2015.	Kinship detection using simulated RNA-seq data from genotypes from **unrelated** individuals (**KHV** population).

	HG02138<br>HG02139<br>HG01840<br>HG01841<br>HG01842<br>HG01843<br>HG01844		
Genotypes	HG01669<br>HG01602<br>HG01670<br>HG01605<br>HG01765<br>HG01766<br>HG01500<br>HG01501<br>HG01503<br>HG01504<br>HG01506	The 1000 Genomes Project Consortium, Nature, 2015.	Kinship detection using simulated RNA-seq data from genotypes from **unrelated** individuals (**IBS** population).
Genotypes	NA19017<br>NA19019<br>NA19020<br>NA19025<br>NA19026<br>NA19027<br>NA19307<br>NA19308<br>NA19309<br>NA19310<br>NA19312	The 1000 Genomes Project Consortium, Nature, 2015.	Kinship detection using simulated RNA-seq data from genotypes from **unrelated** individuals (**LWK** population).
Genotypes	HG01119<br>HG01121<br>HG01122<br>HG01130<br>HG01131<br>HG01142<br>HG01133<br>HG01134<br>HG01136<br>HG01137<br>HG01139	The 1000 Genomes Project Consortium, Nature, 2015.	Kinship detection using simulated RNA-seq data from genotypes from **unrelated** individuals (**CLM** population).
Genotypes	HG00759<br>HG00766<br>HG00844<br>HG00851<br>HG00864<br>HG00867<br>HG00978<br>HG00982<br>HG01028<br>HG01029<br>HG01031	The 1000 Genomes Project Consortium, Nature, 2015.	Kinship detection using simulated RNA-seq data from genotypes from **unrelated** individuals (**CLX** population).
Genotypes	HG01961	The 1000 Genomes	Kinship detection using

	HG01965<br>HG02006<br>HG02102<br>HG02150<br>HG02252<br>HG02262<br>HG02265<br>HG02266<br>HG02274<br>HG02275	Project Consortium, Nature, 2015.	simulated RNA-seq data from genotypes from **unrelated** individuals (**PEL** population).
Genotypes	HG01985<br>HG02051<br>HG02052<br>HG02095<br>HG02111<br>HG02283<br>HG02330<br>HG02332<br>HG02334<br>HG02337<br>HG02339	The 1000 Genomes Project Consortium, Nature, 2015.	Kinship detection using simulated RNA-seq data from genotypes from **unrelated** individuals (**ACB** population).
Genotypes	HG01500<br>HG01501<br>HG01503<br>HG01504<br>HG01506<br>HG01767<br>HG01768<br>HG01785<br>HG01786<br>HG02219<br>HG01507<br>HG01509<br>HG01510<br>HG01512<br>HG01513<br>HG01515<br>HG01516<br>HG01518<br>HG01519<br>HG01521	The 1000 Genomes Project Consortium, Nature, 2015.	Identification of a pair of RNA-seq samples from related individuals in a dataset containing 20 unrelated individuals (**IBS** population).

Supplementary Table 2. The effect of SNP filtering on the range of number of SNPs used for IBD estimation, the true positive rate of genotype calls for RNA-seq samples from individuals NA12877 and NA12878 from the CEPH/UTAH family 1463, the clustering of samples in ternary diagrams and pedigree reconstruction of family 1463 using PRIMUS.

Filter	*minimum number of SNPs used for IBD estimation*	*maximum number of SNPs used for IBD estimation*	*% correctly predicted genotypes (NA12878)*	*% correctly predicted genotypes (NA12877)*	*Incorrect clustering of samples in ternary diagram*	*Pedigree predicted correctly*

None	6248	9901	99.21	98.66	no	yes
Imprinted genes	6227	9877	99.23	98.74	no	yes
Repeats	5680	8621	99.30	98.72	no	yes
Segmental duplications	5638	8929	99.45	98.95	no	yes

Supplementary Table 3. Expected IBD probabilities of different relationships and degrees of relatedness. Z0, Z1 and Z2 represent the probabilities that a pair of individuals share 0, 1 or 2 identical by descent alleles respectively.

Degree	*Relationship*	*Z0*	*Z1*	*Z2*
1	parent-offspring	0	1	0
	full-siblings	0.25	0.5	0.25
2	grandparental, avuncular, half-siblings	0.5	0.5	0
3	First-cousins, great-grandparental, great-avuncular, half-avuncular	0.75	0.25	0
∞	unrelated	1	0	0

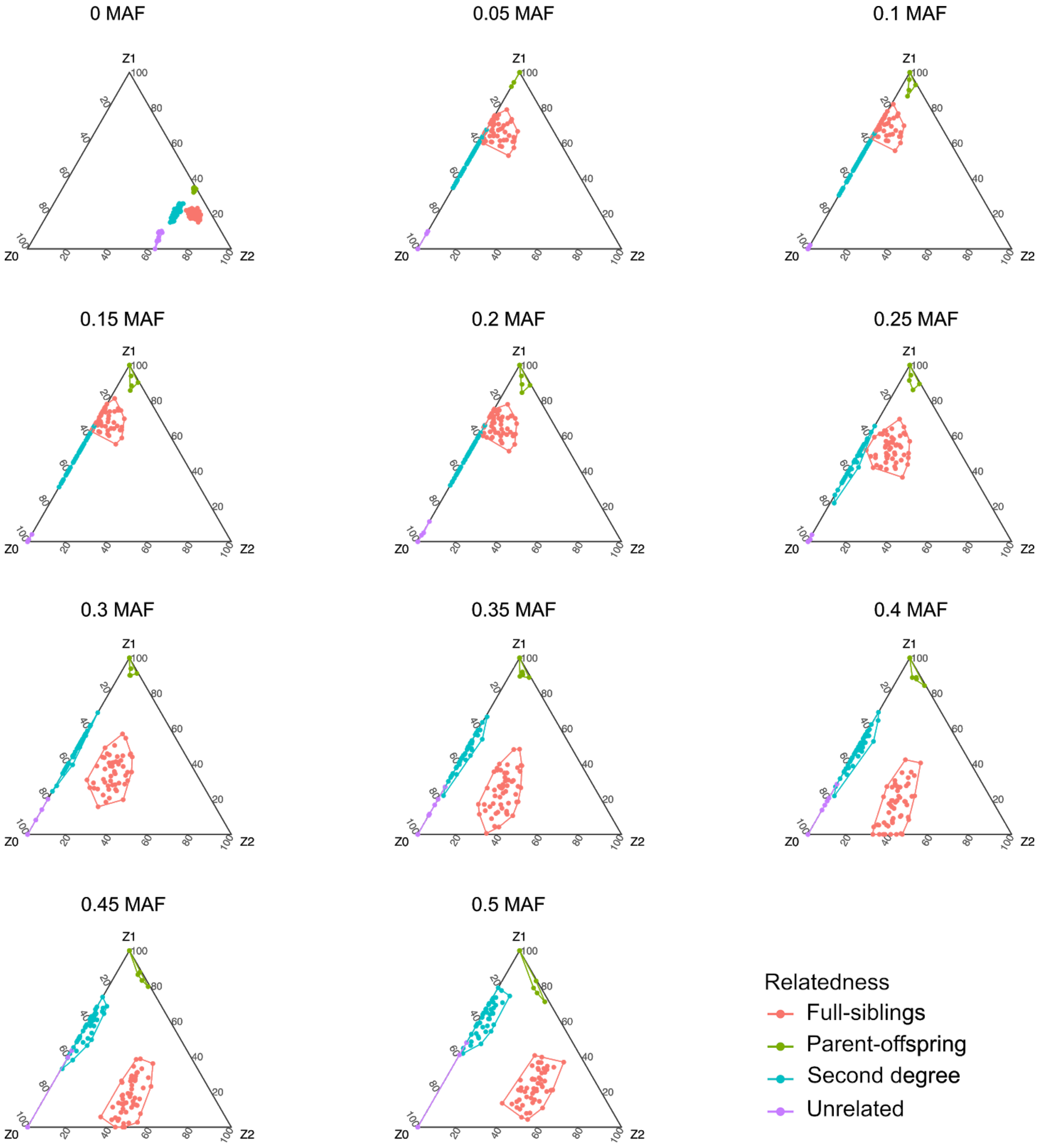

Supplementary Figure 1: Ternary diagrams of IBD estimates for CEPH/UTAH family 1463 using a minor allele frequency threshold ranging from 0 to 0.5.

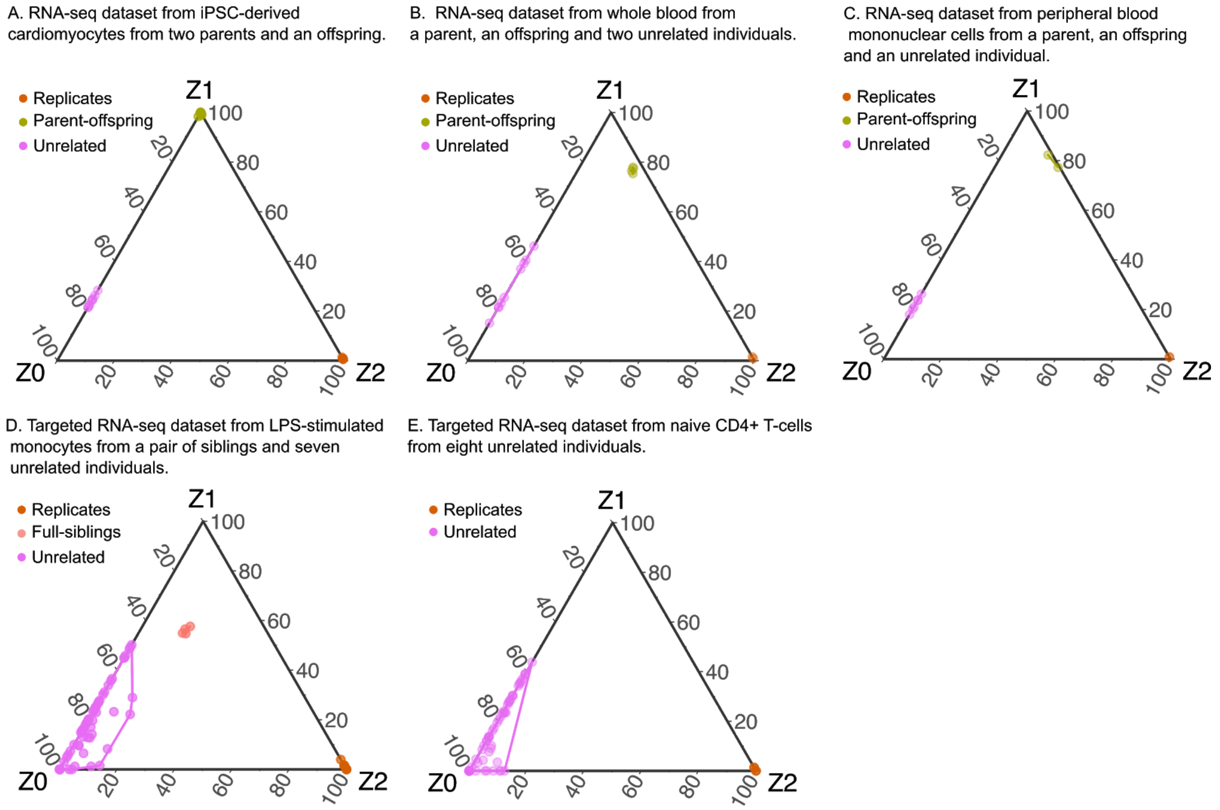

Supplementary Figure 2: Ternary diagrams of IBD estimates from empirical RNA-seq datasets from related and unrelated individuals (A-E). Data from Gifford *et al* Science 2019 (A), Oda *et al* Front Immunol 2019 (B,C) and Boutboul *et al* J Clin Invest 2018 (D,E).

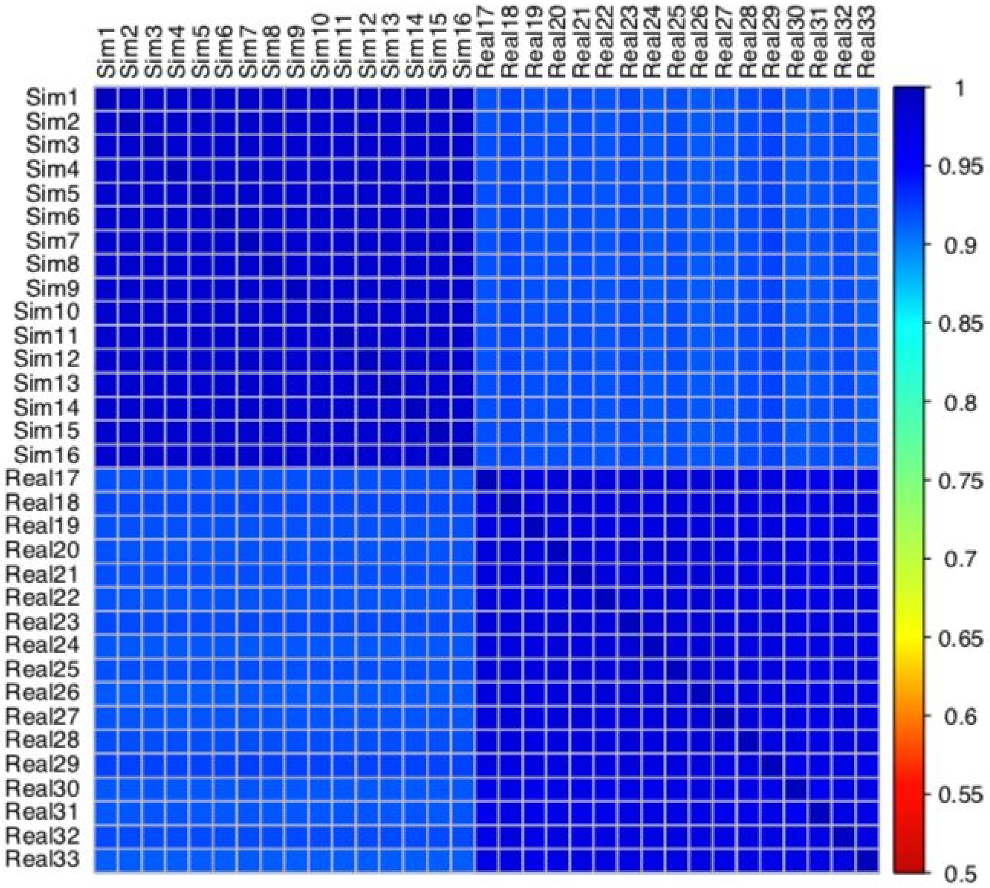

Supplementary Figure 3. Correlation matrix of the number of reads per gene in logarithmic scale between Simulated data (Sim1 - Sim16: individuals 1 - 16 of a type 1 simulated family) and Real data (Real1 - Real17: SRR1258217 - SRR1258233). Mean correlation between Real data: 0.975, between Simulated data: 0.988, between Real data and Simulated data: 0.918.

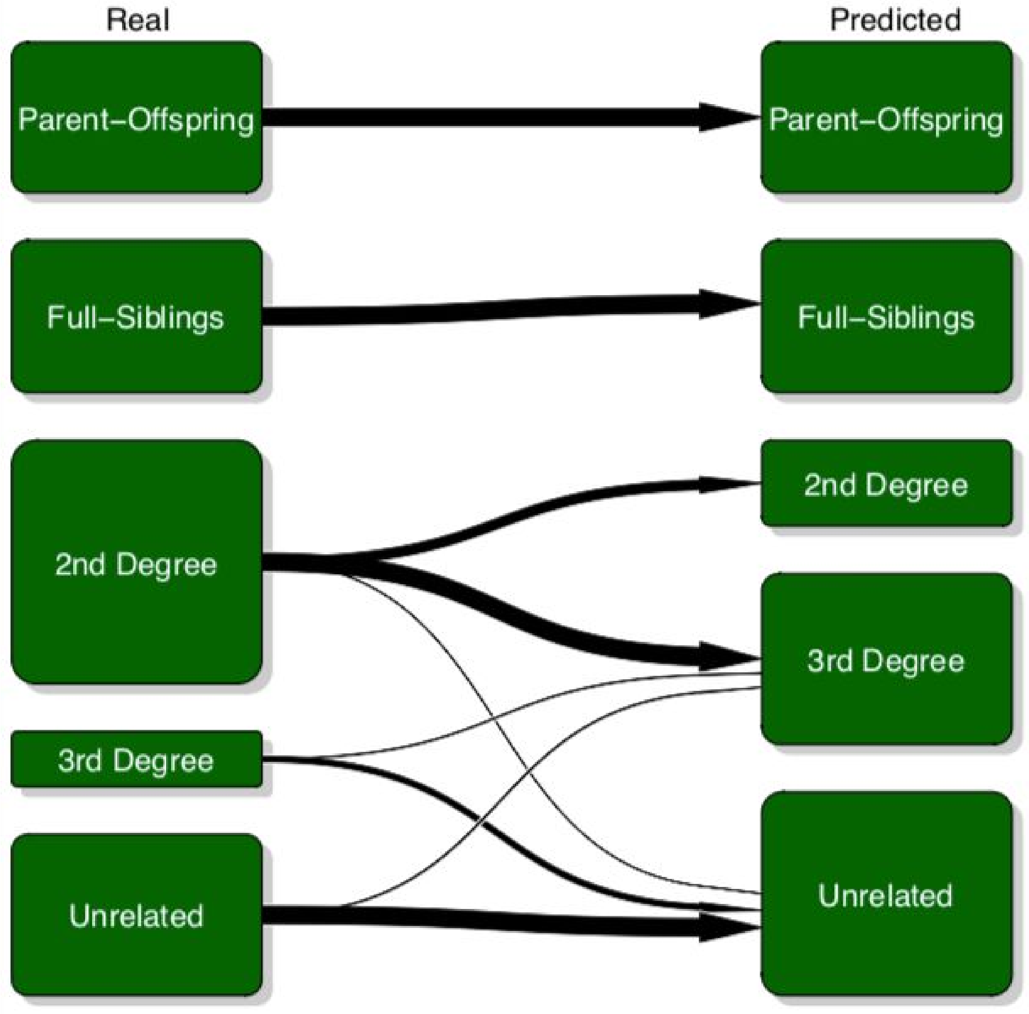

Supplementary Figure 4: Kinship classification by PRIMUS for simulated families (pairs with higher than third degree relationship are represented here as unrelated).

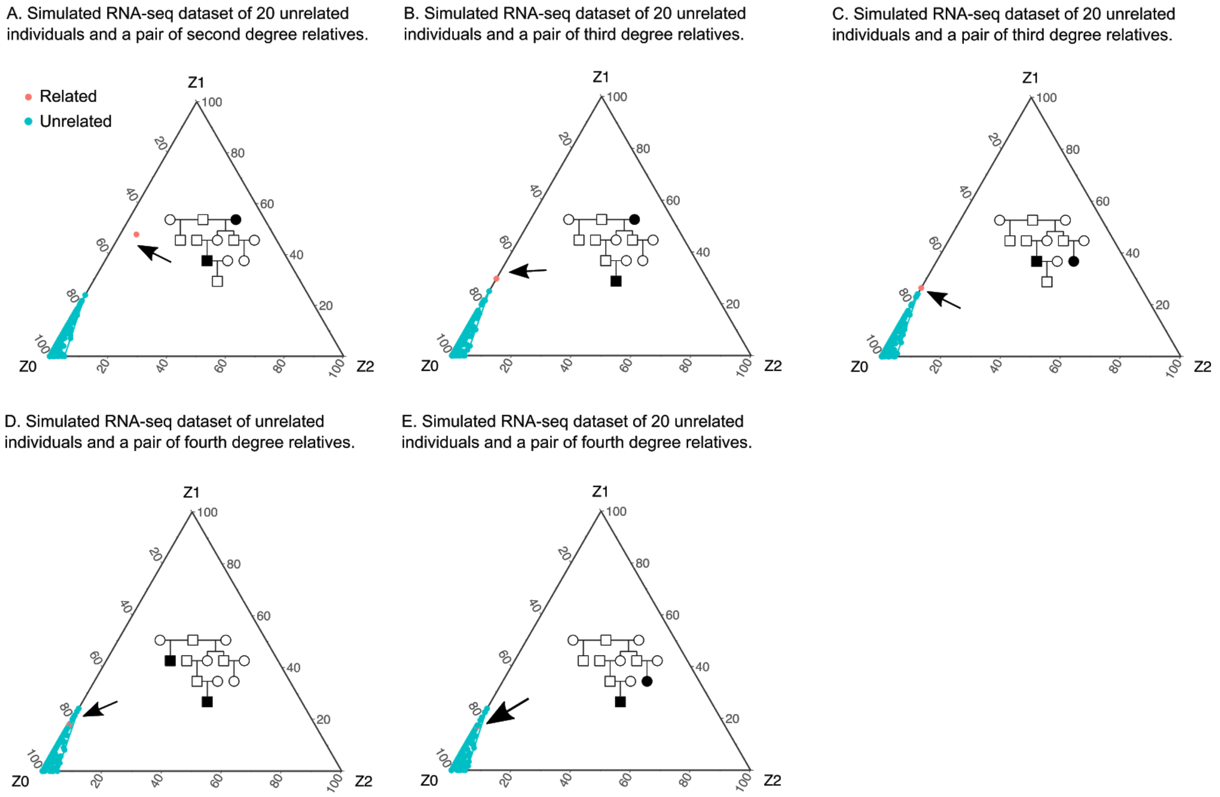

Supplementary Figure 5: Use of kinship detection for identification of RNA-seq samples from related individuals within a larger datasets of samples from unrelated individuals. Ternary diagrams of IBD estimates for simulated RNA-seq samples from twenty unrelated individuals from the IBS population and a pair of related individuals from a type 4 simulated pedigree (A-E). The pair of related individuals from the pedigree used to spike the dataset of 20 unrelated individuals is shown in black in the pedigree inside each ternary diagram.

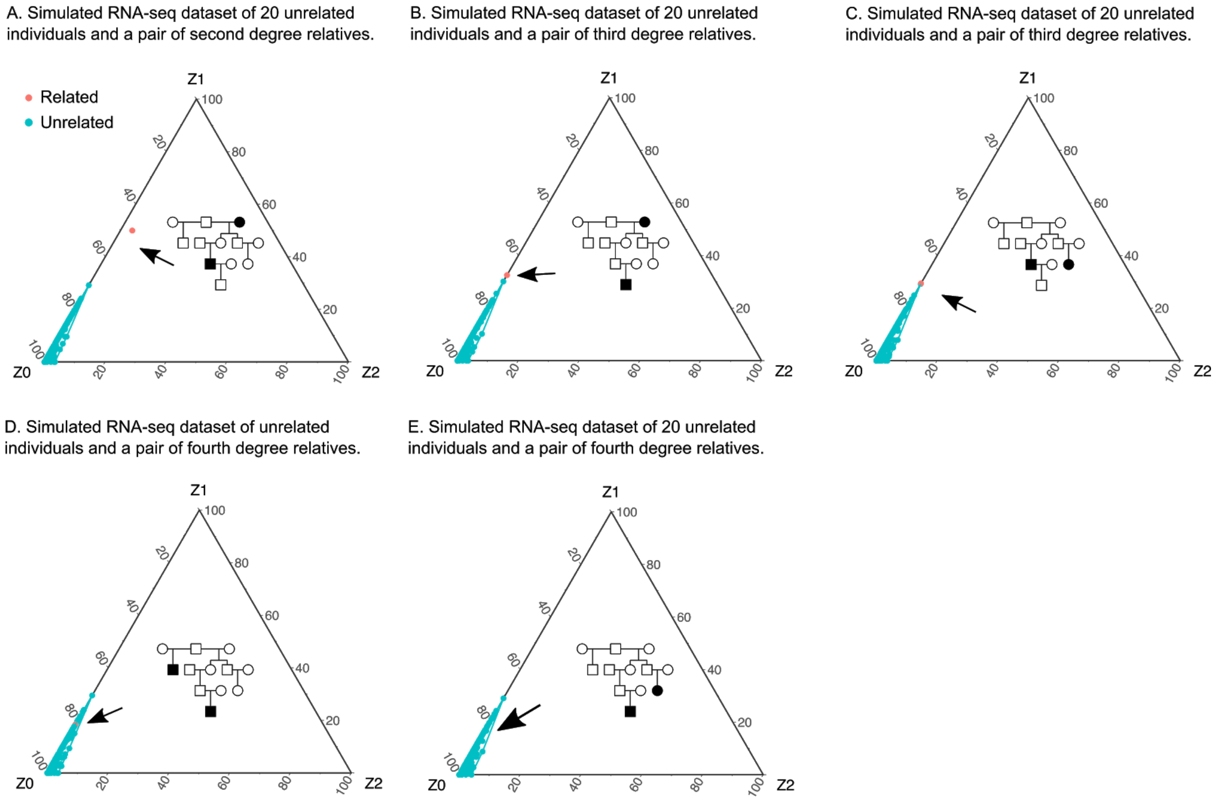

Supplementary Figure 6: Ternary diagrams of IBD estimates from simulated RNA-seq datasets consisting of samples from twenty unrelated and a pair of related individuals from the IBS population (genotypes retrieved from the 1000 Genome Project), after removing variants in imprinted genes, repeats and also human segmental duplications from She et al, Nature 2014. The pedigree and five ternary diagrams (A-E) correspond to the same simulated datasets shown in Supplementary Figure 3.
